# Pathogenicity, colonization, and innate immune response to *Pasteurella multocida* in rabbits

**DOI:** 10.1186/s12917-022-03517-9

**Published:** 2022-11-26

**Authors:** Wenhao Yang, Mingtao Li, Chengcheng Zhang, Xiaorong Zhang, Mengjiao Guo, Yantao Wu

**Affiliations:** 1 Jiangsu Co-Innovation Center for Prevention of Animal Infectious Diseases and Zoonoses, College of Veterinary Medicine, Yangzhou University, Yangzhou, 225009 China; 2 Joint International Research Laboratory of Agriculture & Agri-Product Safety, Yangzhou University (JIRLAAPS), Yangzhou, 225009 China

**Keywords:** *Pasteurella multocida*, Pasteurellosis, Acute septicemic syndrome, Innate immunity, Toll-like receptors

## Abstract

**Background:**

*Pasteurella multocida* (*P. multocida*) infection can cause a series of diseases in different animals and cause huge economic losses to the breeding industry. *P. multocida* is considered to be one of the most significant pathogens in rabbits. In order to elucidate the pathogenic mechanism and innate immune response of *P. multocida*, an infection experiment was carried out in this study.

**Results:**

Our results showed that the clinical symptoms of rabbits were severe dyspnoea and serous nasal fluid. During the course of the disease, the deaths peaked at 2 days post infection (dpi) and mortality rate was 60%. The pathological changes of the lung, trachea, and thymus were observed. In particular, consolidation and abscesses appeared in lung. Histopathologic changes in rabbits showed edema, hemorrhage, and neutrophil infiltration in the lung. *P. multocida* can rapidly replicate in a variety of tissues, and the colonization in most of the tested tissues reached the maximum at 2 dpi and then decreased at 3 dpi. The number of *P. multocida* in lung and thymus remained high level at 3 dpi. Toll-like receptors 2 and 4 signaling pathways were activated after *P. multocida* infection. The expression of *Il1β*, *Il6*, *Il8*, and *Tnf-α* was significantly increased. The expression of most proinflammatory cytokines peaked at 2 dpi and decreased at 3 dpi, and the expression trend of cytokines was consistent with the colonization of *P. multocida* in rabbit tissues.

**Conclusions:**

The *P. multocida* can rapidly replicate in various tissues of rabbit and cause bacteremia after infection. TLRs signaling pathways were activated after *P. multocida* infection, significantly inducing the expression of proinflammatory cytokines, which is might the main cause of respiratory inflammation and septicemia.

## Background

*Pasteurella multocida* (*P. multocida*) is responsible for different diseases in a variety of animals, including farm animals (cattle, pig, rabbit, dog, and chicken), wild mammals, reptiles, and laboratory animals [1]. *P. multocida*-related diseases of huge economic losses to the breeding industry mainly include fowl cholera in avian, haemorrhagic septicemia and bovine respiratory disease in ruminant, porcine progressive atrophic rhinitis and swine plague, and complex respiratory diseases in rabbits [2]. It can also cause respiratory infections in humans, but human deaths are uncommon [3]. Using Carter’s method, *P. multocida* has been serologically divided into five serotypes (A, B, D, E, and F) based on capsular antigens [4, 5]. On this basis, there are 16 serotypes (serovar 1-16) according to lipopolysaccharide antigens [5]. Different serotypes of *P. multocida* are host specific, and can cause different diseases and pathological types among different animal species. Serotypes associated with fowl cholera are generally A:1, A:3, and A:4 [6]; haemorrhagic septicemia is generally caused by serotypes B:2 and E:2 [7]; bovine respiratory disease is frequently induced by serotype A:3 [8]; and porcine progressive atrophic rhinitis is caused by toxigenic serogroups D and A [9]. Pasteurellosis in rabbits is more usually induced by serotypes A. In 2008, *P. multocida* serogroup F was confirmed to be highly pathogenic in rabbits [10]. In Italy, 39 isolates from rabbits affected by different diseases were characterized as serogroup A (20/39), D (9/39), and F (10/39) during 1924–2008 [11].

In rabbits, pasteurellosis is considered one of the most common diseases, and the prevalence rate ranges from 4.3 to 100% [12, 13]. Like most *Pasteurella* species, *P. multocida* is considered to be part of the normal flora of the oropharyngeal. Rabbits are often colonized with *P. multocida* for long periods without clinical symptoms. It is also a primary or opportunistic pathogen of the respiratory tract. Diseases develop when animals are subjected to certain stresses, such as transportation, overcrowding, changes in ambient temperature or humidity. Stresses are the important factor, which seems to be conducive to promote the proliferation and toxicity of microorganisms, and its mechanism is not fully understood [10, 14]. Therefore, the control of *P. multocida-*related diseases is very difficult. Similar to *P. multocida* in various livestock and poultry varieties under intensive production conditions, *P. multocida* also causes high economic losses in rabbits. Although *P. multocida* has been discovered for decades, there are few studies on the pathogenesis of the disease caused by *P. multocida* in rabbits.

*P. multocida* infection in rabbits mainly causes a series of inflammatory diseases and septicemia. Innate immune system is the first line of defense against the invasion of pathogenic microorganisms. Pattern recognition receptors detect pathogen-associated molecular patterns and damage-associated molecular patterns [15]. Among all pattern recognition receptors, toll-like receptors (TLRs) family is one of the most studied families [16]. TLR2 and TLR4 are located on the cell surface and can recognize pathogen-associated molecular patterns of different pathogen surface molecules. After activation, TLRs recruits downstream effectors, triggers downstream signaling cascades, and induces proinflammatory cytokines and chemokines, such as IL-1β, IL-6, IL-8, IL-12, TNF-α, and IFN-γ. IL-1β is a major proinflammatory cytokine that mediates innate immunity and plays a key regulatory role in the response to infection and injury. IL-1β can enhance the production of TNF-α, IL-6, and IL-8 [17]. In the process of innate immune response, proinflammatory cytokines can induce immune cells to migrate to the inflammatory site and eliminate pathogens. In turn, different types of leukocytes, including natural killer cells and mononuclear macrophages, secretes proinflammatory cytokines, promotes lymphocyte proliferation and differentiation, and participates in inflammatory response [18]. Although disease caused by *P. multocida* has been reported in rabbits, study on the role of host innate immune response against *P. multocida* infection are scarce. In this study, we investigated the colonization of *P. multocida*, pathological changes, clinical symptoms, and innate immune responses in rabbits after *P. multocida* infection*.*

## Materials and methods

### Pathogen

The *P. multocida* C51-17 strain (serovar A) was cultured on sheep blood agar plates supplemented with 5% sheep red blood cells at 37 °C for 24–36 h. The *P. multocida* colony was grown in trypticase soybean broth supplemented with 5% fetal bovine serum at 37 °C shaker for 12 h. According to the results of viable count, the bacterial culture was diluted in PBS to 10^6^ colony forming unit per mL (CFU/mL) for the challenge experiment.

### Animals and challenge experiment

A total of 50 healthy weaned New Zealand White rabbits (35 days old) used in this study were purchased from a commercial rabbitry and housed individually in stainless steel rabbit cages, which were placed in isolated rooms with independent ventilation. The rabbits were randomly separated into two groups and free access to water and feed. Each group was housed in a separate room. In order to confirm the absence of *P. multocida* infection, nasal and conjunctival swabs were collected from all rabbits before challenge. The swabs were plated onto sheep blood agar plates at 37 °C for 24–36 h for bacterial examination. The swab samples were also detected by 16S rRNA gene which is species-specific for *P. multocida* (Table 1). Challenge experiment was performed after 3 days of feeding. The challenge dose and route was determined in preliminary experiment. The challenge experiment was conducted by using subcutaneous injection with 1 mL of *P. multocida* bacterial suspension (10^6^ CFU/mL). Rabbits of the control group were injection with 1 mL of PBS.

**Table 1:** Primers sequences used in this study

Primer name	Sequence(5′-3′)
qPm-F	GGATGTTGTTAAATAGA
qPm-R	TAGACTCCCAGTCTGAA
q*Tlr2*-F	GCTGCGCAAGATCATGAACA
q*Tlr2*-R	TTTATGGCGGCCCTCAAGTT
q*Tlr4*-F	AGGCTGTTGGTGGAAGTTGA
q*Tlr4*-R	TGCTTATCTGACAGGTGGCA
q*Il1β*-F	TGGCACGTATGAGCTGAAAG
q*Il1β*-R	GGCCACAGGTATCTTGTCGT
q*Il6*-F	CTGAAGACGACCACGATCCA
q*Il6*-R	AAGGACACCCGCACTCCAT
q*Il8*-F	CTCTCTTGGCAACCTTCCTG
q*Il8*-R	TTGCACAGTGAGGTCCACTC
q*Tnf-α*-F	CACTTCAGGGTGATCGGC
q*Tnf-α*-R	TGCGGGTTTGCTACTACG
q*Gapdh*-F	AGGTCATCCACGACCACTTC
q*Gapdh*-R	GTGAGTTTCCCGTTCAGCTC

Ten rabbits were selected to calculate the mortality and clinical symptoms scores independently by assigning random numbers (Microsoft Excel, Microsoft Corporation). The attitude, temperature, and chest murmur, incidence, and deaths were observed and counted daily. Clinical symptoms were scored separately from 0 to 3 (Table 2). The survival rate was calculated at the end of the challenge experiment. At 1, 2, and 3 days post infection (dpi), five rabbits were selected from each group. The moribund rabbits were killed in extremis. Others were selected by assigning random numbers and euthanized by bleeding from jugular vein under the anesthesia with sodium pentobarbital (30 mg/kg body weight) for necropsy and subsequent research, including histopathological examination, RNA and DNA extraction.

**Table 2:** The scoring criteria for clinical symptoms

Scores	Respiratory symptoms	Diarrhea
0 (normal)	Eyes and nose is clean with no discharge, normal breathing	No diarrhea, the fur around perianal area and tail is dry and clean
1 (mild)	Serous discharge from eyes or nose	Soft to loose stool, no fecal material adhering to fur
2 (moderate)	Mucopurulent nasal discharge, increased respiratory rate	Loose stool or mixed with mucus, liquid fecal material adhering to fur
3 (severe)	Excessive mucopurulent nasal discharge, tachypnea, pulmonary murmur	Projectile watery stool with mucus, fecal material covering large portions of the perianal area, hind limbs, and tail.

### Histopathological changes

The thymus, lung, and trachea of 5 rabbits in each group were collected at 1, 2, and 3 dpi to observe histopathological changes. The tissues were fixed with 4% paraformaldehyde solution at room temperature. The fixed tissue samples were embedded in paraffin and then sectioned at 5 μm. Next, the sections were stained with hematoxylin and eosin (H&E). Histopathological changes were observed using a Leica DM2000 LED light microscope (Leica, Wetzlar, Germany).

### *P. multocida* quantification

The lung, liver, thymus, spleen, heart, and kidney of 5 rabbits in each group were collected to investigate the colonization of *P. multocida* at 1, 2, and 3 dpi*.* DNA was extracted with the Bacteria Genomic DNA kit (CWbio, Beijing, China) and stored at − 20 °C until further use. Quantitative real-time PCR (qRT–PCR) reaction system was carry out in a total volume of 20 μL with *TransStart*^R^ Tip Green qPCR SuperMix (+Dye II) (Transgen Biotech Co., Ltd., Beijing, China). The primers qPm-F and qPm-R were designed for the detection of *P. multocida* (Table 1). qRT–PCR was performed with the LineGene 9600 Plus real-time PCR System (Bioer Technology, Hangzhou, China). The qRT–PCR program involved 1 cycle of 94 °C for 30 s, followed by 40 cycles of 94 °C for 5 s and 60 °C for 34 s and finally followed by a dissociation curve. Make three replicates for each sample.

### Transcriptional levels of innate immunity-related genes

The lung and thymus of 5 rabbits in each group were collected to investigate the transcriptional levels of innate immunity-related genes at 1, 2, and 3 dpi*.* Total RNA was extracted from the samples using TRIzon reagent RNA kit (CWbio). RNA (1 μg) was reverse transcribed with TransScriptR one-step gDNA Removal and cDNA Synthesis SuperMix (Transgen Biotech Co., Ltd.). The synthesized cDNA was stored at − 20 °C. For qRT–PCR, the primers for *Tlr2*, *Tlr4*, *Il1β, Il6*, *Il8*, *Tnf-α,* and *Gapdh* were based on the previous studies (Table 1) [19, 20]. qRT–PCR reaction system was carry out in a total volume of 20 μL with *TransStart*^R^ Tip Green qPCR SuperMix (+Dye II) (Transgen Biotech Co., Ltd.). qRT–PCR was performed with the LineGene 9600 Plus real-time PCR System (Bioer Technology). The qRT–PCR program involved 1 cycle of 94 °C for 30 s, followed by 40 cycles of 94 °C for 5 s and 60 °C for 34 s and finally followed by dissociation curves. The housekeeping gene *Gapdh* was used as the internal control to normalize the target genes. The relative mRNA transcriptional levels of target genes were calculated using the 2^−ΔΔCt^ method. The results were expressed as the fold change of target gene expression in infected rabbits (*n* = 5) vs. that of the control group (n = 5). Make three replicates for each sample.

### Statistical analysis

The statistical analysis was performed by SPSS 23.0 software (SPSS Inc., Chicago, IL). The difference of survival rate of rabbits was analyzed using the Kaplan–Meier method. Other data were analyzed with the Student’s *t*-test. A value of *p <* 0.05 was set as statistical significance.

## Results

### Clinical symptoms and survival rate

**Fig. 1:**
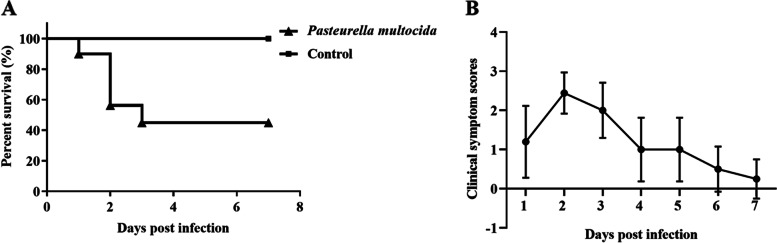
The survival rate and clinical symptoms of rabbits after challenge with *P. multocida*. **A** Rabbits were challenged by subcutaneous injection of 1 mL *P. multocida* bacterial suspension (10^6^ CFU/mL). Kaplan–Meier method was used to examine difference of survival rate. **B** The clinical symptom scores of rabbits after challenge

Before challenge, no *P. multocida* was detected in the nasal and conjunctival swabs, indicating that all rabbits were free of *P. multocida* infection. No clinical symptoms and deaths were observed in control group during the experiment. Depression, fever, loss of appetite, shortness of breath, and serous nasal discharge occurred at 1 dpi, of which 2/10 rabbits showed moderate diarrhea. As shown in Fig. 1A, the mortality rate was 10% at 1 dpi. Diarrhea was relieved at 2 dpi, but severe dyspnoea with excessive mucopurulent nasal discharge. The average clinical symptoms score reached a maximum of 2.4 at 2 dpi (Fig. 1B). The deaths peaked at 2 dpi and ultimately reached 60% at 3 dpi. The diseased rabbits suffered from acute septicemia syndrome, which ended with severe dyspnoea and shock. The clinical symptoms were relieved at 3 dpi. No deaths occurred thereafter.

### Necropsy lesions

**Fig. 2:**
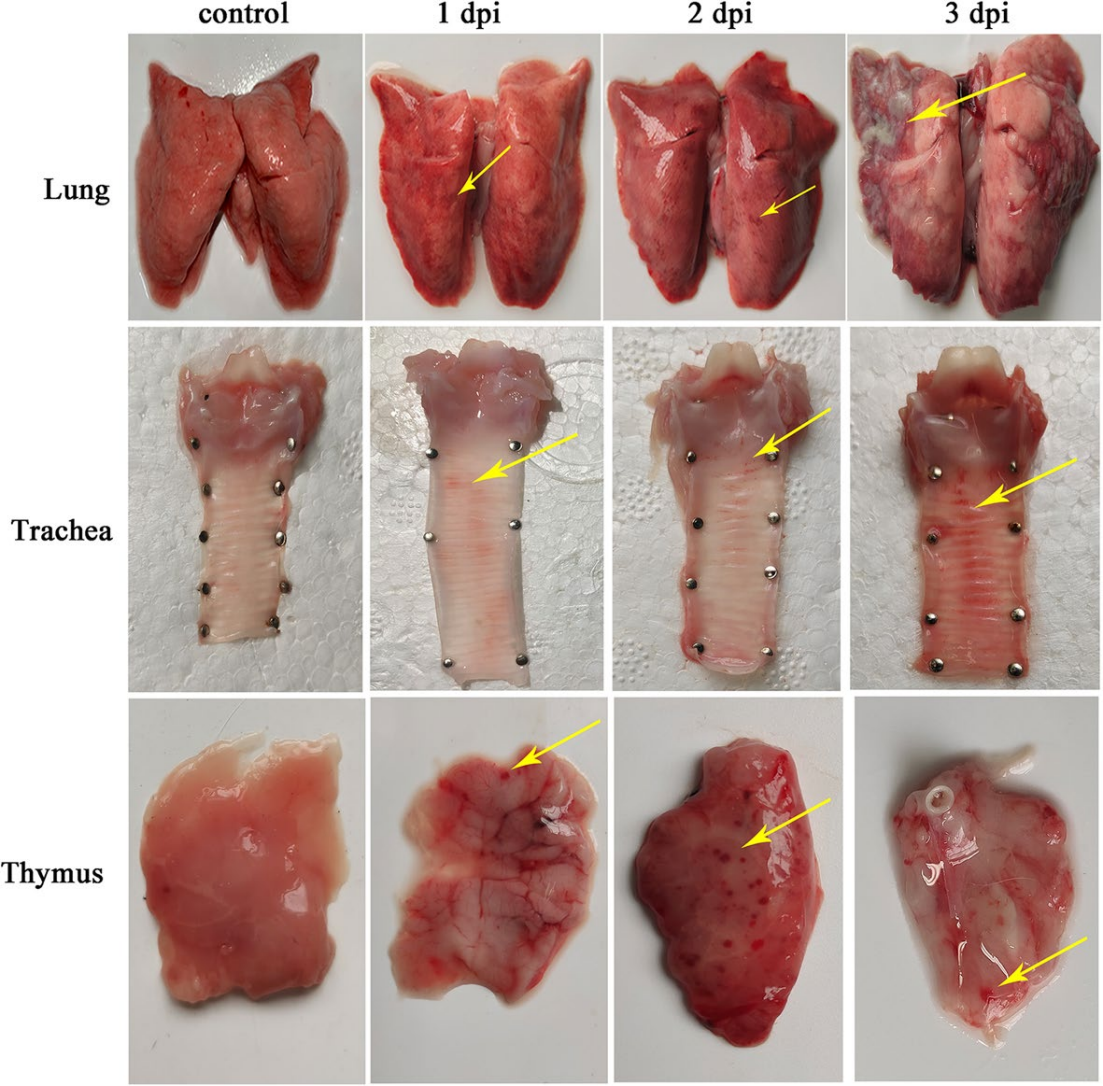
Necropsy lesions of rabbits after challenge with *P. multocida*. At 1, 2, and 3 dpi, the necropsy lesions in lung, trachea, and thymus were observed. Consolidation and abscesses were observed in lung. Hemorrhage was observed in trachea and thymus

No gross lesions of the disease were observed in rabbits of control group. The pathological changes were mainly focus on the lung, with slight bleeding and consolidation at 1 and 2 dpi. The lungs developed typical consolidation at 3 dpi, with abscesses and fibrous exudates. Hemorrhage occurred in the tracheal ring after challenge, most severe at 3 dpi. Similarly, the thymus exhibited obvious bleeding spots, which are the most severe at 2 dpi (Fig. 2).

### Histopathological analysis

**Fig. 3:**
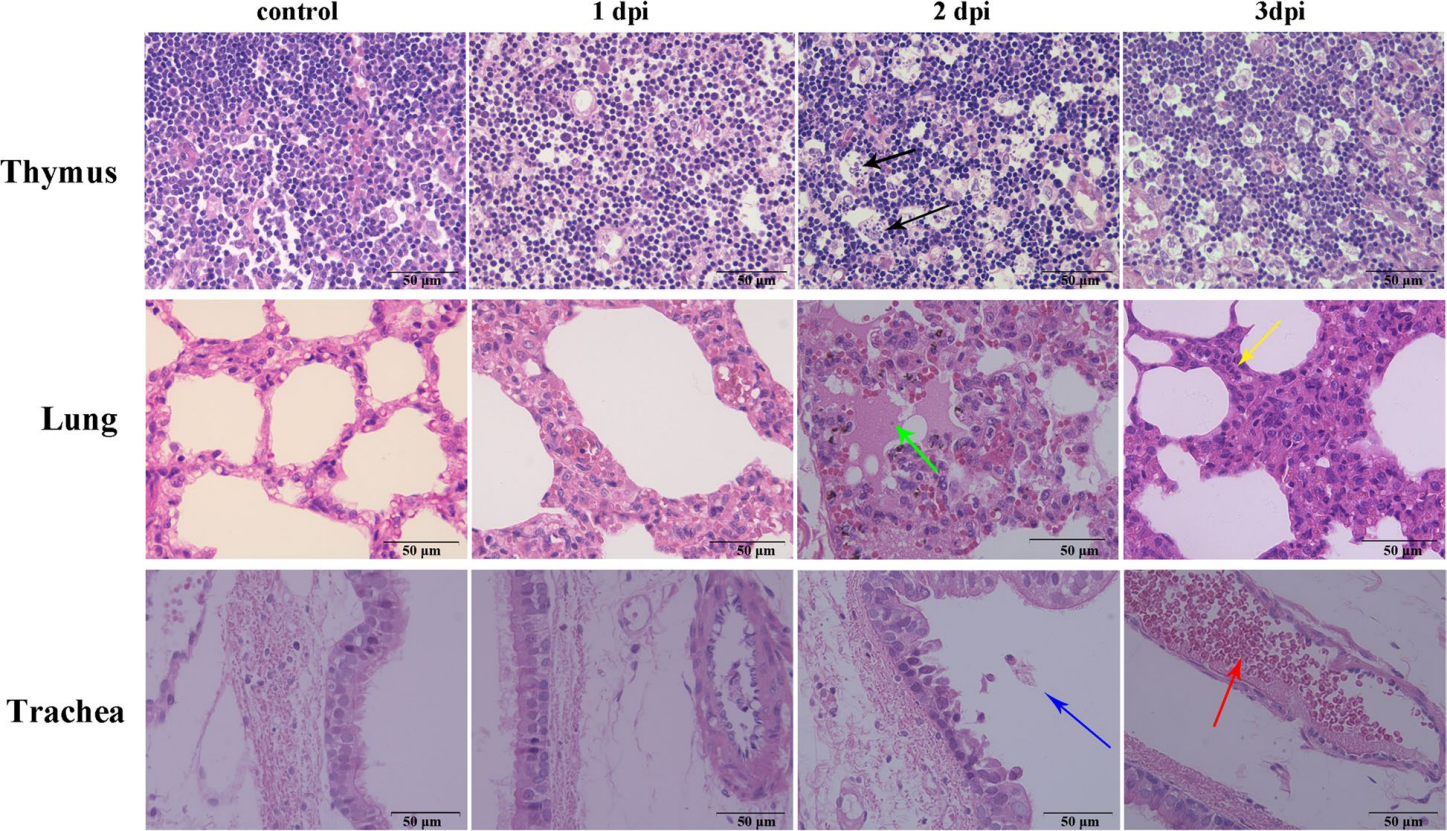
Histopathologic changes in rabbits after challenge with *P. multocida*. The number of lymphocytes decreased and cell necrosis in thymus (black arrow). Edema, hemorrhage (green arrow), and neutrophil infiltration (yellow arrow) in the lung. Necrotic, shed (blue arrow), congestion (red arrow) in the trachea

As shown in Fig. 3, hispathological changes occurred in thymus, lung, and trachea in the infected rabbits. At 1 dpi, the number of lymphocytes decreased in the thymus. No obvious pathological changes were observed in lung and trachea. On the second day after challenge, the tissue damage was aggravated. The number of lymphocytes decreased and cell necrosis occurred in thymus. Edema and hemorrhage was observed in the lung. The epithelial cells of tracheal mucosa were necrotic and shed. At 3 dpi, the number of lymphocytes continued to decrease and a large number of cells were necrotic. Neutrophil infiltration was observed in the lung. The tracheae exhibited congestion. There were no pathological changes in rabbits of control group. Combined with clinical symptoms and gross lesions, *P. multocida* infection in rabbits caused pathological damage to the lungs and trachea.

### *P. multocida* content in tissues of infected rabbits

**Fig. 4:**
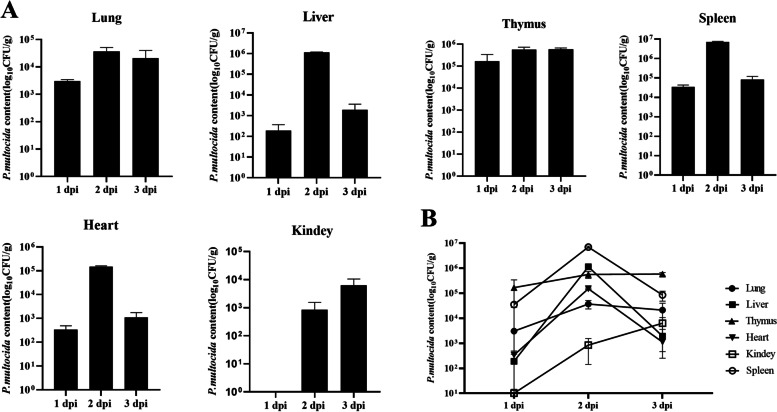
The contents of bacteria in different tissues of rabbits after challenge with *P. multocida*. **A** *P. multocida* content (log_10_ CFU/g) in lung, liver, thymus, spleen, heart, and kidney of infected rabbits. **B** Trend curve of *P. multocida* content at 1, 2, and 3 dpi. Bars represented the means ± SD (*n* = 5)

As shown in Fig. 4A, the bacterial contents were relatively low at 1 dpi, and no *P. multocida* were detected in kidney. The thymus had the highest number of *P. multocida* at 1.67 × 10^5^ CFU/g, followed by the spleen. *P. multocida* was detected in all tissues at 2 dpi. The bacterial content in the spleen reached 6.96 × 10^6^ CFU/g at 2 dpi, which was the highest among all tissues. Then the bacterial count in the spleen dropped at 3 dpi. Changes in bacterial load in the liver and heart were similar to those in the spleen, peaking at 2 dpi, followed by a dramatic decline at 3 dpi (Fig. 4B). The bacterial counts in the liver and heart dropped to 1.91 × 10^3^ CFU/g and 1.11 × 10^3^ CFU/g at 3 dpi, respectively. However, the number of *P. multocida* in the lung and thymus increased gradually from 1 to 3 dpi. The number of *P. multocida* in the thymus increased to 5.59 × 10^5^ CFU/g at 2 dpi and remained the highest at 3 dpi. These results suggested that *P. multocida* can rapidly invade the host, reach and replicate in various tissues, causing tissue damage and even death.

### Expression of toll-like receptors after challenge with *P. multocida*

**Fig. 5:**
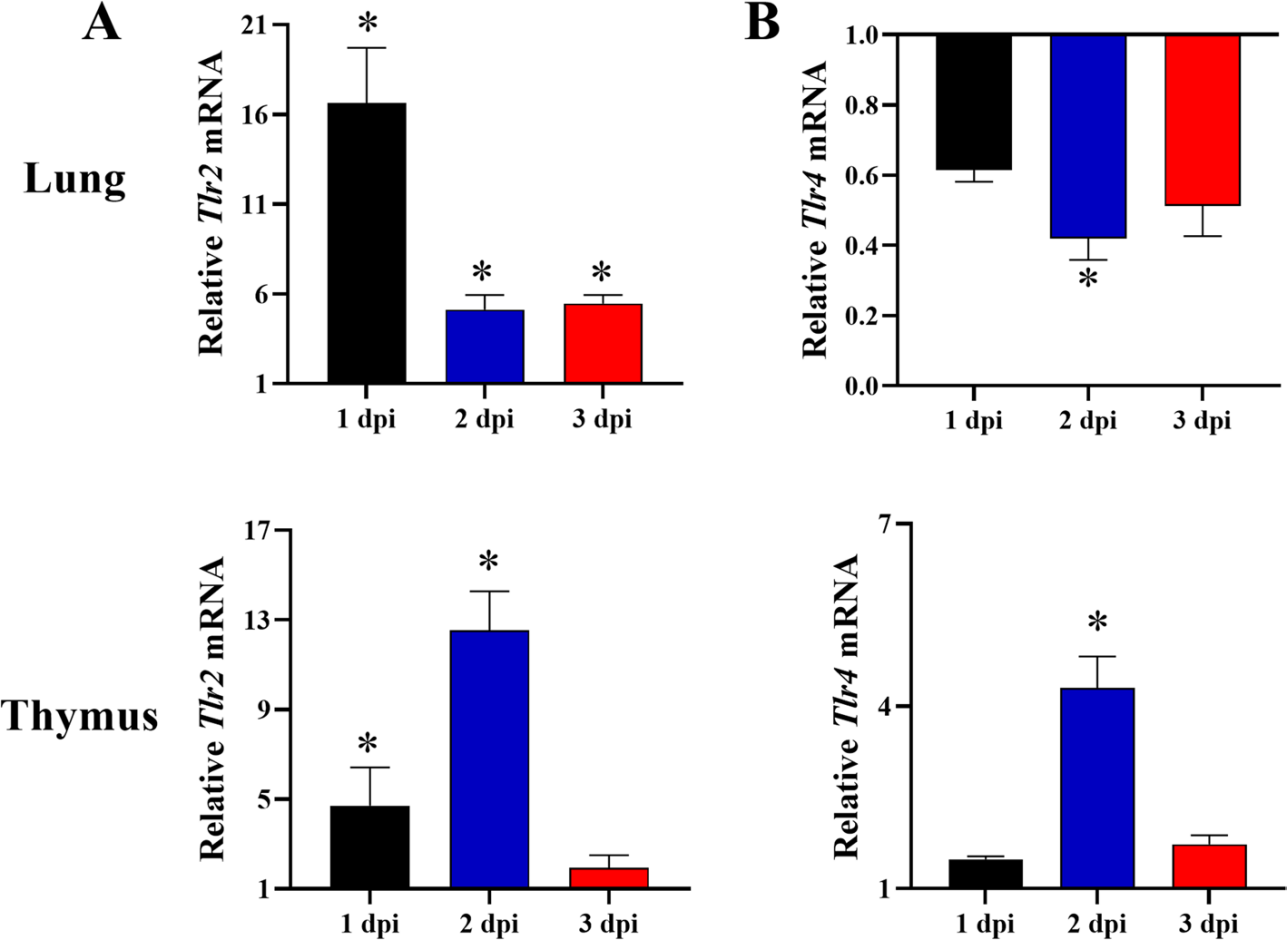
The expression of Toll-like receptors mRNA in *P. multocida-*infected rabbits. The expression of (**A**) *Tlr2* and (**B**) *Tlr4* in lung and thymus at 1, 2, and 3 dpi. Relative mRNA of target gene was calculated from the infected rabbits compared with that of the control group. Bars represented the means ± SDs (*n* = 5). *p < 0.05

Innate immunity is a conserved host defense system activated by the pattern recognition receptors. As shown in Fig. 5A, in tested tissues, the expression of *Tlr2* was significantly up-regulated at 1 dpi, especially in the lung by 16.63-fold (*p <* 0.05). The expression of *Tlr2* reached the peak at 2 dpi in thymus, significantly up-regulated by 12.53-fold (*p <* 0.05). The expression trend of *Tlr4* in thymus was similar to that of *Tlr2*, significantly up-regulated by 4.30-fold at 2 dpi (*p <* 0.05) and only a 1.72-fold increase at 3 dpi. However, the expression of *Tlr4* was suppressed in the lung, especially by a significant 0.42-fold at 2 dpi (Fig. 5B). These results indicated that the pattern recognition receptors pathways were activated after challenge and that the expression of *Tlr4* was tissue-dependent.

### Expression of Proinflammatory cytokines after challenge with *P. multocida*

**Fig. 6:**
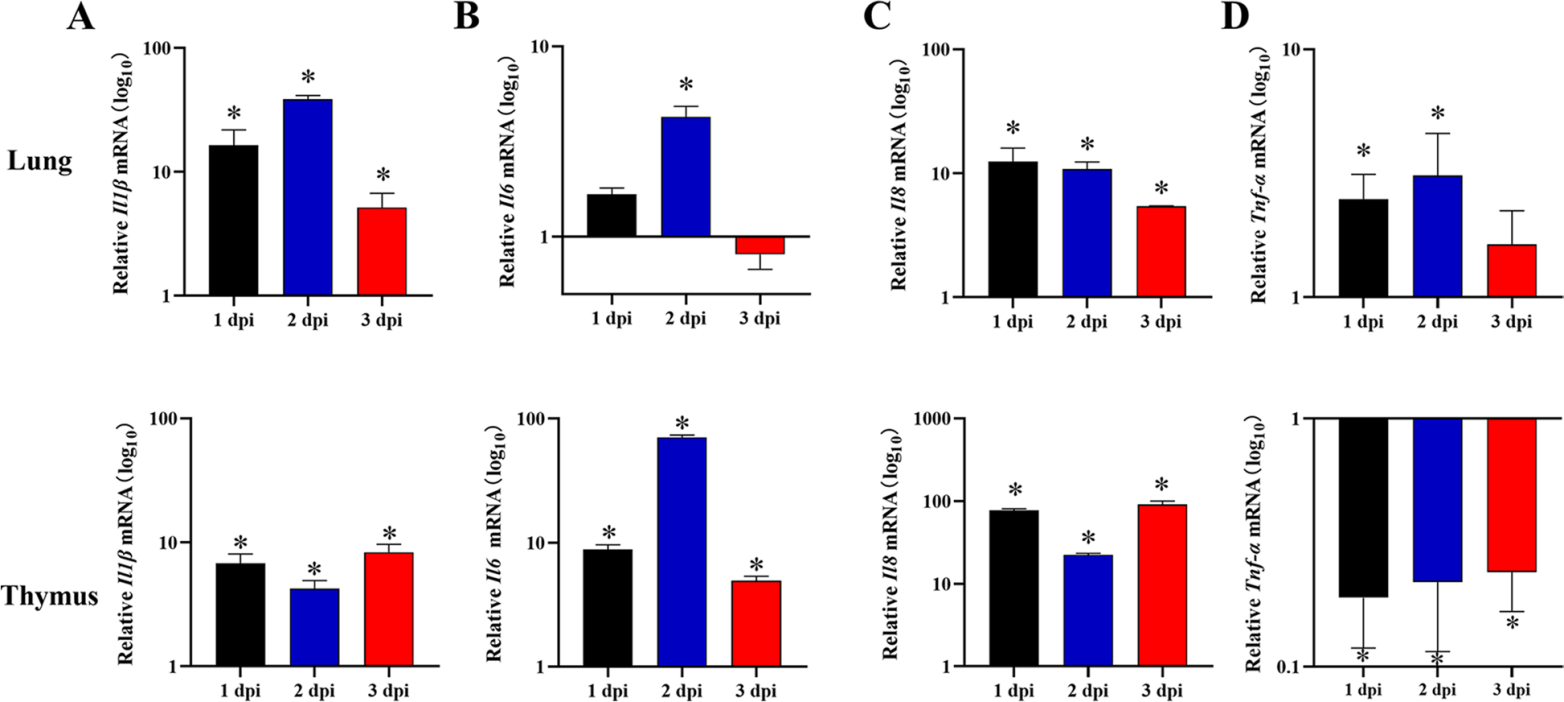
The expression of inflammatory factors mRNA in *P. multocida-*infected rabbits. The expression of (**A**) *Il1β*, (**B**) *Il6*, (**C**) *Il8*, and (**D**) *Tnf-α* in lung and thymus at 1, 2, and 3 dpi. Relative mRNA of target gene was calculated from the infected rabbits compared with that of the control group. Bars represented the means ± SDs (*n* = 5). *p < 0.05

After TLRs recognizes bacteria and activates, it triggers downstream signal cascade and induces proinflammatory cytokines. The expression of most cytokines peaked at 2 dpi and decreased at 3 dpi. As shown in Fig. 6A, *Il1β* was significantly up-regulated in lung and thymus. The expression of *Il1β* was the highest in lung, which was significantly increased by 38.48-fold at 2 dpi (*p <* 0.05). Similarly, the expression of *Il6* was the highest at 2 dpi, up-regulated 4.26-fold in the lung and 70.81-fold in the thymus (*p <* 0.05) (Fig. 6B). The expression of *Il8* was significantly up-regulated in lung and thymus at 1, 2, and 3 dpi. It was the highest among all proinflammatory cytokines, up-regulated 77.22-fold and 91.31-fold in the thymus at 1 and 3 dpi, respectively (*p <* 0.05). In lung, the expression of *Il8* significantly up-regulated 12.46-fold at 1 dpi, then gradually decreased, and up-regulated 5.41-fold at 3 dpi (*p <* 0.05) (Fig. 6C). However, there was a remarkable difference in the expression of *Tnf-α* and other proinflammatory cytokines. It was up-regulated 2-3-fold in lung, but significantly down-regulated in thymus (Fig. 6D).

## Discussion

Pasteurellosis is widely distributed all over the world and can spread across species and regions [21]. As the number of rabbits used as food, household pets, and laboratory animals increases, as well as the increase of the number and activities of wild rabbit populations, the risk of interspecific pathogen transmission has also increased. In this study, we examined the clinical symptoms, histopathological changes, colonization of *P. multocida*, and host innate immune responses in *P. multocida-*infected rabbits.

The clinical symptoms of pasteurellosis in rabbit include rhinitis with purulent nasal discharge, pneumonia, abscesses, otitis media, and septicemia [22–24]. In current study, clinical symptoms were observed at 1 dpi. The infected rabbits presented primarily with respiratory symptoms, with severe dyspnoea and serous nasal fluid. At the end of the experiment, the mortality rate was 60%. Our results were similar with previous study, 11/12 rabbits were seriously ill. All the sick rabbits developed acute septic syndrome with respiratory failure and shock [10]. Previous research showed that the inflammation caused by *P. multocida* due to the infiltration of neutrophils and macrophages in lungs of buffalo calves [25]. In this study, rabbits showed respiratory symptoms after infection, with the obvious necropsy lesions and histopathological changes in the lungs. Consolidation, hemorrhage, and neutrophil infiltration were observed in the lung. Hemorrhage was observed in trachea and thymus. The thymus is the largest immune organ in rabbit. In thymus, the number of lymphocytes decreased and cell necrosis occurred. The pathogenicity of *P. multocida* is directly related to its colonization in rabbit tissues. Our results showed that, with the exception of the thymus and kidney, the proliferation of *P. multocida* in tested tissues reached a maximum at 2 dpi and then declined at 3 dpi. The mortality rate of rabbits was also the highest at 2 dpi. In addition, the number of *P. multocida* in lung and thymus remained high level at 3 dpi, which was consistent with the results of pathological changes. According to the above results, it was revealed that *P. multocida* can rapidly replicate in various tissues and organs of rabbit and cause bacteremia after infection.

Innate immunity system is the first line of defense against the infection of pathogenic microorganisms, which is mediated by phagocytes such as dendritic cells and macrophages. TLR4 binds to the co-receptors MD-2 and CD14 of lipopolysaccharide of Gram-negative bacteria to recognize it in soluble form or on bacteria [26]. TLR2, together with TLR1 or TLR6, identifies pathogen-associated molecular patterns as homodimers or heterodimers [27]. In this study, the expression of *Tlr2* and *Tlr4* was significantly induced in thymus after challenge with *P. multocida*. The expression of *Tlr2* significantly up-regulated by 16.63-fold in lung. Our results indicated that TLR2 signaling pathway was activated after *P. multocida* infection. According to previous study, *P. multocida* infection promote the expression of pattern recognition receptors, inflammatory cytokines, and chemokines in murine lungs. Especially, up-regulation of TLR2 and NLRs mediates *P. multocida* infection [28]. However, other studies reported that the association of TLRs in *P. multocida* infection is mainly that TLR4 mediated signal pathway recognizes the toxin and LPS [29, 30]. Our results showed that the expression of *Tlr4* significantly increased in thymus, but inhibited in lung. Therefore, we speculated that *P. multocida* can activate TLR4-mediated signaling pathways to induce inflammatory responses, but in a tissue-dependent manner. On the other hand, excessive proinflammatory cytokine production can lead to severe immunopathological changes, even life-threatening. For this reason, in the development of inflammation, a feedback system is required to regulate the restriction and balance between the proinflammatory and anti-inflammatory.

In mice and cattle, the inflammatory mediators were highly expressed in lung after *P. multocida* infection. The number of neutrophils and the expression of proinflammatory cytokines (TNF-α, IL-6, IL-1, IL-8, and IL-12) increased in lung [31]. These inflammatory responses play important roles in *P. multocida* infections. After activation of TLRs, NF-κB signaling was stimulated to resist bacterial infection by regulating production of cytokines [32, 33]. Recently, previous study has been demonstrated that the expression of pro-IL-1β induced by *P. multocida* is partially dependent on TLR4 in macrophages. The expression of pro-IL-1β was significantly up-regulated after *P. multocida* infection, but partially decreased in TLR4 knockout macrophages [34]. In current study, the expression of *Il1β* was significantly increased in lung and thymus. IL-6 and IL-8 are important cytokines in the inflammatory response, and cooperate with other cytokines to regulate the inflammatory response process. TNF-α is an endogenous pyrogen that induces fever and stimulates endothelial cells and leukocytes to release various inflammatory mediators that promote neutrophil phagocytosis. It has been reported that *P. multocida* highly induced the secretion of proinflammatory cytokines (TNF-α, IL-1β, and IL-6) in mouse [35]. Similarly, our results showed that the expression of *Il6*, *Il8*, and *Tnf-α* was significantly increased, indicating that the TLRs pathway was activated after *P. multocida* challenge. The expression of most cytokines peaked at 2 dpi and decreased at 3 dpi. This trend was consistent with the colonization of *P. multocida* in rabbit tissues. We speculated that the high expression of proinflammatory cytokines (*Il1β*, *Il6*, *Il8*, and *Tnf-α*) in lung might be the main cause of respiratory inflammation and septicemia.

In conclusion, the whole disease process of *P. multocida* infection in rabbit were investigated. The clinical symptoms were mainly respiratory symptoms, with severe dyspnoea and serous nasal fluid. The pathological changes are directly related to colonization of *P. multocida* in rabbit tissues. TLRs signaling pathways were activated after *P. multocida* infection, the overexpression of inflammatory factors aggravates tissue damage and mortality, which is also the main cause of respiratory inflammation and septicemia. Altogether, this study has pioneered the systematic exploration of the colonization, innate immune responses, and etiology in *P. multocida*-infected rabbits. This study also provide new insights into the relationship between *P. multocida* pathogenicity and defense response in rabbits.

## Data Availability

The datasets used and/or analysed during the current study available from the first author (E-mail: yangwenhao1991@163.com) on reasonable request.
